# Aflatoxins and Human Health: Global Exposure, Disease Burden, and One Health Strategies

**DOI:** 10.3390/toxins18020090

**Published:** 2026-02-10

**Authors:** Jill Koshiol, Amit Yadav, John D. Groopman, Usha Dutta

**Affiliations:** 1 Division of Cancer Epidemiology and Genetics, National Cancer Institute, Rockville, MD 20874, USA; 2 Department of Gastroenterology, All India Institute of Medical Sciences, New Delhi 110029, India; amity1994.ay@gmail.com; 3 Department of Environmental Health and Engineering, Johns Hopkins Bloomberg School of Public Health, Baltimore, MD 21205, USA; 4 Department of Gastroenterology, Postgraduate Institute of Medical Education and Research, Chandigarh 160012, India

**Keywords:** aflatoxin, aflatoxin B_1_, aflatoxin–albumin adducts, aflatoxin–lysine albumin adducts, gallbladder cancer, hepatocellular cancer, One Health, prevalence, cancer, prevention

## Abstract

Mycotoxin contamination represents a major public health and economic burden worldwide. Aflatoxins, particularly aflatoxin B_1_, are the most detrimental for human health. In this review, we discuss the sources of exposure and geographic distribution. The prevalence of aflatoxin–albumin/lysine adduct detection in humans varies dramatically across the world, from 0% reported in two European studies to up to 100% reported in studies from parts of Africa and Asia. We also summarize the disease outcomes that aflatoxins are associated with in humans. We focus particularly on cancer outcomes, which aflatoxins can cause through mutagenic DNA adducts, oxidative stress, mitochondrial dysfunction, immune effects, and epigenetic changes. Synergy with hepatitis B virus and potentially with other mycotoxins can also increase risk. Minimization of aflatoxin exposure requires an integrative approach, beginning at the farm level and continuing through pre-harvest, post-harvest, storage, and the consumer level. New developments in technology, such as electrochemical biosensors and artificial intelligence algorithms, are being piloted and could help improve detection and decontamination efforts. Further, new tests for aflatoxin exposure in humans (e.g., blood spot assays) could assist biomonitoring efforts. Despite regulatory standards in most countries for the maximum allowable level of aflatoxins in food products and animal feed, exposure remains high in many parts of the world and might be increasing even in countries with historically low exposure. Integration of these tools in a One Health framework is essential to reduce the current and future burden of aflatoxin-related disease.

## 1. Introduction

Mycotoxins are secondary metabolites produced by certain kinds of fungi [1] that can accumulate in food because of fungal contamination and lead to human exposure from farm to table unless safe and regulated practices are followed throughout the food supply chain [2]. These toxins are invisible to the naked eye, lack a characteristic odor, and do not alter the taste of food, making their detection extremely difficult. Identification of mycotoxins requires specific and sophisticated analytical methods, which are often cost-intensive [3]. Food contamination can occur at any stage of production, ranging from the pre-harvest period to storage within households. Importantly, mycotoxins are not completely destroyed by routine food-processing techniques such as baking, frying, boiling, cooking, or pasteurization [4]. They retain their chemical structure and carcinogenic potential; therefore, regulatory authorities must ensure that mycotoxin levels are minimized before food products reach consumers’ homes. Minimizing mycotoxin contamination requires a holistic, comprehensive, and integrated One Health approach that spans the entire food system, from primary production at the farm level to final consumption.

Mycotoxins cause a considerable human disease burden and have a major detrimental economic impact through contamination of crops. Globally, it has been estimated that anywhere from 25% to over 80% of agricultural products are contaminated with mycotoxins [5,6,7,8,9]. While mycotoxins such as ochratoxin A, zearalenone, deoxynivalenol, and fumonisins have been noted to affect human health, aflatoxins are among the most studied with respect to human disease [10].

Although there are many kinds of mycotoxins, aflatoxins have been of particular interest because of their known toxicities and their wide-spread prevalence as contaminants in food products and livestock feed [11]. Aflatoxin is a well-established liver carcinogen [12], with an estimated 17% (14–19%) of hepatocellular carcinomas (HCC) attributable to aflatoxin [13]. It has been estimated that ~4.5 billion people live in latitudes with particularly high risk of chronic exposure [14]. This estimate is based on a 1999 report, however. While updated prevalence data are needed, the high risk of exposure in these regions and documented diseases associated with aflatoxin exposure has led to a strong regulatory focus on aflatoxin exposure. For these reasons, this review focuses largely on aflatoxins.

Aflatoxins were discovered in the 1960s through investigation of “turkey X” disease, leading to identification of aflatoxins as contaminants of poultry feed [15,16,17]. Around the same time, aflatoxin was associated with hepatoma in rainbow trout [18]. Although there are seven different kinds of fungi that produce aflatoxins, *Aspergillus flavus* and *Aspergillus parasiticus* produce the majority of aflatoxins, including aflatoxin B_1_, aflatoxin B2, aflatoxin G_1_, and aflatoxin G_2_ [11,16]. The name “aflatoxin” is a combination of the “A” in *Aspergillus* with the “fla” in flavus plus “toxin” [16]. The first toxic secondary metabolite identified as a product of *Aspergillus flavus* had a blue fluorescence blue, leading to the name “aflatoxin B,” while the second fluoresced green, leading to the name “aflatoxin G” [16]. Aflatoxins designated as “M” are metabolites of aflatoxin B_1_ found in milk [19]. The numbers (B_1_, B_2_, G_1_, etc.) reflect the relative distance traveled on a thin-layer chromatographic plate [20].

Although aflatoxin B_1_ is the most studied aflatoxin, important gaps remain in understanding its pathogenic mechanisms [11]. The spectrum of aflatoxin-associated diseases in humans has not been fully defined. In addition, biomonitoring efforts and global efforts to focus on One Health have lagged behind.

## 2. Sources of Exposure

### 2.1. Environmental Reservoirs

Soil serves as the primary ecological reservoir for aflatoxin-producing *Aspergillus* species. Fungal spores persist in soil and crop residues, contaminating agricultural commodities either directly during crop growth or indirectly during harvesting, drying, and storage. Environmental stressors such as drought, elevated temperatures, and insect damage promote fungal colonization and activate aflatoxin biosynthetic pathways. The long-term persistence of toxigenic *Aspergillus* strains in soil explains the recurrent nature of aflatoxin contamination across successive growing seasons [21]. Among environmental factors, temperature and humidity are the most critical determinants, with optimal aflatoxin production occurring at temperatures of 25–35 °C and water activity levels above 0.83 [7,22]. However, these factors also interact with agronomic practices such as irrigation and storage to influence realized environmental contamination. In addition, fungal persistence and exposure risk can be modified by soil management practices, which is explored in more detail in Section 6.1. In tropical and subtropical regions, inadequate storage conditions further exacerbate this risk, particularly in small retail outlets and household storage systems where ventilation and moisture control are often insufficient.

### 2.2. Plant-Based Food Sources

Human exposure occurs largely through the consumption of contaminated foods, making aflatoxins a paradigmatic example of diet-mediated environmental carcinogens. The food commodities most frequently implicated in aflatoxin exposure are plant-derived staples and condiments that are either highly susceptible to fungal growth or consumed in large quantities.

Maize has consistently emerged as the dominant contributor to dietary aflatoxin exposure at the global level. Although maize does not always exhibit the highest concentrations of aflatoxins when compared with nuts or spices, its central role as a staple food in many regions results in a disproportionately high contribution to total daily intake [23]. Extremely high aflatoxin B_1_ concentrations in maize have been documented in sub-Saharan Africa and Central America, with reported levels reaching several thousand micrograms per kilogram in poorly regulated settings. In high-consumption settings such as rural Kenya and Tanzania, maize consumption exceeding 400–600 g per person per day, even with mean aflatoxin concentrations in the range of 10–100 µg/kg, results in daily intakes well above 5 µg aflatoxin B_1_ per person. Maize samples have shown extreme contamination, with reported aflatoxin B_1_ concentrations ranging from several hundred micrograms per kilogram to a maximum exceeding 9000 µg/kg, underscoring the dominant role of maize both in chronic exposure and in acute aflatoxicosis episodes [24]. However, these very high contamination values represent outlier cases and are not representative of typical contamination levels.

Groundnuts represent another major dietary source of aflatoxin exposure and are particularly important in South Asia, Africa, and parts of China. Their high lipid content, combined with direct contact with soil during growth and harvesting, creates an environment conducive to *Aspergillus* colonization. Groundnuts frequently contain aflatoxin concentrations exceeding regulatory limits by several orders of magnitude, and contamination often persists through processing into peanut butter, oils, and confectionery products [25]. In populations where groundnuts are consumed regularly, either as snacks or as ingredients in traditional dishes, they may contribute between one-fifth and two-fifths of total daily aflatoxin intake, depending on consumption patterns and contamination levels. A study suggests that groundnut samples frequently exceed regulatory limits, with total aflatoxin concentrations commonly ranging from 100 to more than 1000 µg/kg, and occasional values surpassing 10,000 µg/kg [26].

Rice has traditionally been regarded as a relatively low-risk cereal for aflatoxin contamination; however, growing evidence from tropical Asia challenges this assumption. Literature shows aflatoxin B_1_ concentrations in rice range from non-detectable levels to approximately 361 µg/kg in Indian samples and up to 185 µg/kg in Sri Lankan samples [27,28]. Although the mean contamination levels in rice are often lower than those observed in maize or groundnuts, the sheer quantity of rice consumed on a daily basis means that it can contribute a large proportion of overall exposure. In rice-based diets, especially in South Asia, rice may account for nearly half of total dietary aflatoxin intake, underscoring the importance of considering consumption volume alongside contamination concentration when assessing exposure risk.

Wheat is a major staple crop, and aflatoxin contamination poses a significant public health concern, particularly in developing countries. Wheat is relatively less prone to aflatoxin contamination than other cereal grains, but it is still an important staple in many regions and can contribute to dietary exposure to aflatoxin under poor post-harvest conditions. Because wheat is consumed daily in large quantities in the form of flour, bread, and other processed foods, even low-level aflatoxin contamination can contribute substantially to chronic dietary exposure [29]. Contamination is often uneven and not detectable by visual inspection, allowing affected grains to enter the food chain. A study conducted in Egypt showed that nearly one third of the wheat grain samples were contaminated; however, among these, only 16.7% exceeded the safe limits [30]. In another study, which assessed aflatoxin B_1_ contamination in 1646 wheat grain samples from rural and urban areas across 10 Indian states, aflatoxin B_1_ levels ≥ 5 µg/kg were detected in 40.3% of samples, while 16% exceeded the Indian regulatory limit of 30 µg/kg [28].

Spices, tree nuts, and dried fruits constitute a unique category of aflatoxin-contaminated foods. Commodities such as dried chilies, turmeric, and black pepper have been reported to contain some of the highest aflatoxin concentrations recorded in food surveillance programs, occasionally exceeding 10,000 µg/kg. These extreme levels are attributed to prolonged drying periods, exposure to open air during processing, and inadequate storage conditions. Tree nuts and dried fruits, including pistachios, almonds, hazelnuts, and figs, are also frequently contaminated with aflatoxins and are major drivers of food safety alerts in international trade. While these foods tend to exhibit high contamination frequencies, their contribution to total dietary aflatoxin intake is usually limited in the general population due to lower consumption volumes [31].

**Figure 1:**
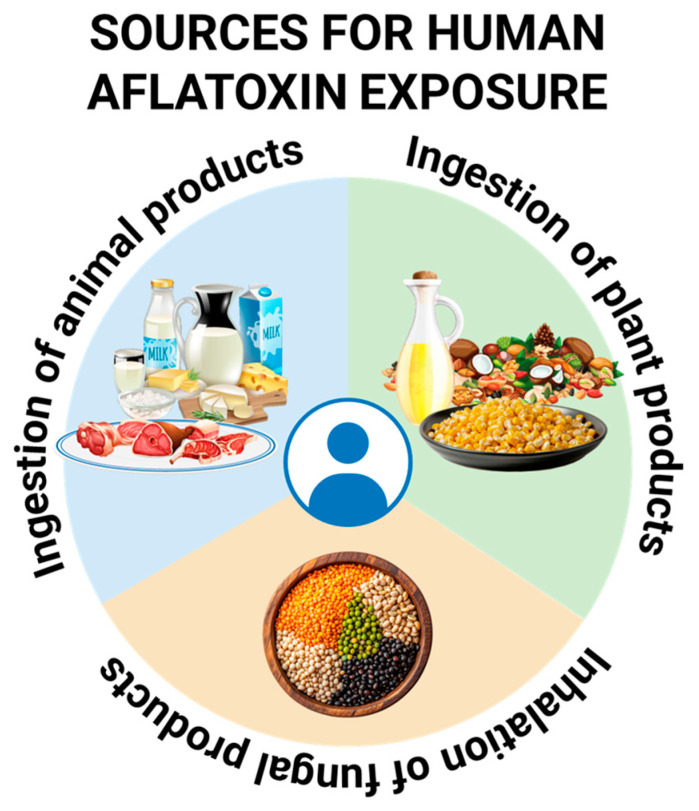
Overview of the major sources of aflatoxin exposure in humans. Aflatoxins produced by *Aspergillus* species contaminate plant-based foods such as maize, groundnuts, cereals, tree nuts, and spices, leading to direct dietary intake. Indirect exposure occurs through consumption of animal products (e.g., milk, meat, and dairy products) when livestock ingest aflatoxin-contaminated feed, resulting in the presence of aflatoxin metabolites such as aflatoxin M_1_. In addition, occupational or environmental exposure may occur via inhalation of aflatoxin-contaminated dust during crop handling, storage, or processing. Together, these pathways contribute to the overall burden of aflatoxin exposure in human populations.

The reasons why certain foods are more heavily contaminated than others are multifactorial and include both intrinsic and extrinsic determinants. Intrinsic characteristics such as high oil content, kernel structure, and susceptibility to physical damage influence fungal growth, while extrinsic factors such as temperature, humidity, drought stress, insect infestation, and storage conditions play a decisive role in determining contamination levels [32]. Processing methods may further distribute aflatoxins within food products, particularly during grinding, which can lead to uniform contamination of flour or paste derived from a small number of highly contaminated kernels (Figure 1).

### 2.3. Animal Sources

Animal-derived foods contribute minimally to total aflatoxin exposure, but they remain relevant from a public health perspective, particularly in children. Aflatoxin M_1_, a hydroxylated metabolite of aflatoxin B_1_, occurs in milk and dairy products as a result of contaminated animal feed. Although aflatoxin M_1_ is less potent as a carcinogen than aflatoxin B_1_, its presence in milk is concerning because milk is consumed regularly by infants and young children [33]. In most dietary assessments, milk contributes less than five percent of total aflatoxin intake, but this contribution may be proportionally higher in pediatric populations. Most countries that regulate aflatoxin M_1_ in milk adopt a maximum limit of 0.5 µg/kg and even stricter limits for infant formula (e.g., 0.025 µg/kg in the EU; see Section 7 on regulatory frameworks). Despite these regulations, non-compliance is frequently reported in low- and middle-income countries due to limited feed monitoring, climatic variability, and heterogeneous enforcement. Animal feed represents a pivotal intervention point for aflatoxin control, as contaminated feed directly leads to aflatoxin M_1_ contamination in milk and dairy products. Meat derived from animals fed contaminated feed contributes to a lesser extent, as aflatoxin accumulation in muscle tissue is relatively low. The carry-over of aflatoxin B_1_ into meat and muscle tissueis substantially lower (<1% of dietary intake) than the carry-over of aflatoxin M_1_ into milk (2–6%) [34,35]. Thus, milk is the most relevant animal-derived exposure pathway, particularly for infants and young children. Nevertheless, chronic dietary exposure through animal-derived foods remains epidemiologically significant [36].

### 2.4. Inhalational Exposure

Inhalation of aflatoxin-contaminated dust may occur in occupational settings such as grain handling, milling, and feed processing. While this route is less relevant for general dietary exposure, it may contribute to localized respiratory or systemic exposure in high-risk occupational groups (e.g., farmers, livestock feed production workers) [37,38,39]. In contrast to oral exposure, dose via inhalation remains poorly characterized, and current data do not allow quantitative risk estimation for lung cancer, which is discussed in later sections.

## 3. Geographic Distribution of Aflatoxins

Growth of aflatoxin-producing fungi is highly influenced by temperature and humidity. Among environmental factors, temperature and humidity are the most critical determinants (see Section 2.1 above). Crops produced in latitudes from 40° N to 40° S of the equator can be particularly susceptible given the temperature and humidity levels in these latitudes [14]. Sub-Saharan Africa bears the highest global burden, driven by heavy reliance on maize and groundnuts combined with hot, humid conditions and limited post-harvest infrastructure [40]. South Asia, particularly India, represents another high-risk region where rice, groundnuts, spices, and milk collectively shape exposure patterns. Monsoon-associated humidity, traditional sun-drying practices, and informal food distribution networks contribute to persistent contamination in this setting [41]. In contrast, Europe and other high-income regions experience relatively low population-level exposure.

However, aflatoxin contamination has been increasing in southern and northern Europe, potentially due to increases in temperature and humidity [42]. Aflatoxin contamination could also come from imported foods since global commerce can introduce contaminated food products into regions without domestic aflatoxin contamination [12]. Furthermore, extreme weather conditions like drought, the fungal genotype, competition from other molds and microorganisms, soil characteristics, and insect damage can all influence susceptibility [14,42].

The seroprevalence of aflatoxin–albumin adduct detection can be affected by the year and the time of year the sample is collected, as aflatoxin exposure can vary by year and across season. Furthermore, detectable versus non-detectable prevalence values have limited comparability across region and time since measurements are sensitive to assay detection limits and cut offs. The prevalence of aflatoxin–albumin adduct detection does not directly translate to dose since some regions with high prevalence may still have low mean adduct levels. In addition, aflatoxin abatement efforts and changes in policy can lead to changes in exposure to aflatoxin over time. For example, in Qidong, China, economic changes facilitating a transition from a corn-based to a rice-based diet led to a dramatic reduction in circulating aflatoxin B_1_–albumin adduct levels [43] from 100% of the population having detectable levels in 1989 to only 7% having detectable levels in 2012 [44]. In addition, the prevalence of aflatoxin–albumin adducts in controls from case–control studies might not be representative of the general population as controls could be matched with cases who have cancer, liver cirrhosis, or other conditions, and they could have underlying disease conditions themselves. Controls might also be drawn from age-restricted populations, such as children or older adults, although one study in The Gambia and Kenya found similar detection prevalences and levels of aflatoxin–albumin adducts in children and adults [45]. In addition, aflatoxin–albumin adducts measured through ELISA may include non-aflatoxin B_1_–lysine adducts (e.g., adducts with aflatoxin G_1_) [46,47]. Despite these limitations, studies of circulating aflatoxin–albumin adducts can provide insight into human exposure to aflatoxins across the globe.

**Figure 2:**
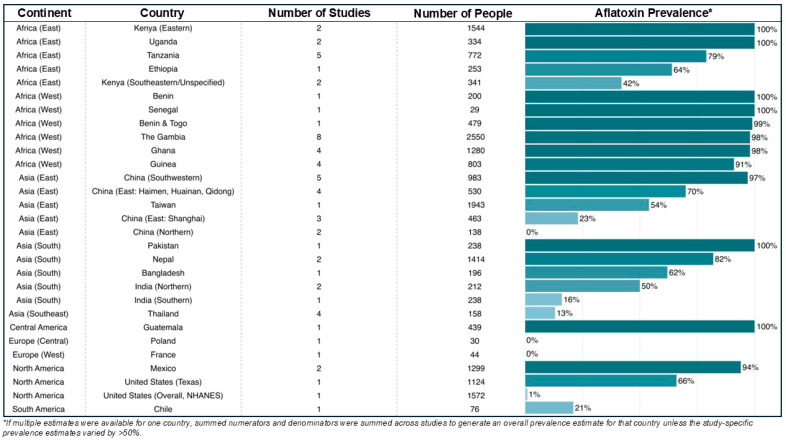
Worldwide prevalence of detectable versus non-detectable circulating aflatoxin–albumin/aflatoxin–lysine adducts.

To gain such insight, we conducted a literature search in PubMed for studies of the detection versus non-detection of circulating aflatoxin–albumin adducts (i.e., aflatoxin–albumin adducts measured in blood samples) in humans. All assays and age groups were included. Most sampling frames were included, although we did exclude an estimate from the Childhood Acute Illness and Nutrition Network [48] since samples from acutely ill children might skew the prevalence upward due to aflatoxicosis. Results for individual studies are provided in Supplemental Table S1, and we summarize results across country in Figure 2. If multiple prevalence estimates were available per country and those estimates differed by less than 50%, we calculated a simple pooled prevalence (i.e., the sum of numerators from each contributing study divided by the sum of the denominators from each contributing study). If estimates within a country differed by more than 50%, we calculated pooled prevalences for regions that had more similar prevalence estimates (e.g., Eastern Kenya vs. Southeastern/Unspecified Kenya) or presented individual study results for nationally representative versus region-specific estimates. If there were multiple publications from the same study population, we chose the prevalence estimate that was based on the largest numbers or the most recent estimate. We excluded studies that did not provide the prevalence of circulating aflatoxin–albumin adduct detection versus non-detection (i.e., if a study provided data on the level circulating aflatoxin–albumin adducts but did not explicitly state that all individuals had detectable values, it was excluded; we did not infer that the prevalence of detection was 100%).

The detection of circulating aflatoxin–albumin adducts exhibits dramatic variation across the world (Figure 2, Supplemental Table S1). West African countries demonstrated a very high prevalence of aflatoxin exposure: 91–100% of tested individuals had detectable levels of aflatoxin–albumin adducts [49,50,51,52,53,54,55,56,57,58,59,60,61,62,63,64,65,66]. The prevalence was also high in East African countries [51,67,68,69,70,71,72,73,74,75,76,77], although it was more variable, even within a country. For example, the prevalence was 100% in two studies from the eastern part of Kenya [69,71], but only 42% in the southeast [51,70] (Supplemental Table S1). Only one study of 24 people from Egypt represented North Africa and found a detectable aflatoxin–albumin adduct prevalence of 100% [78]. In Asia, South Asia had the highest prevalence and again demonstrated notable within-country prevalence in India [79,80,81,82,83,84,85], where the prevalence ranged from 16% in Southern India to 50% in Northern India. In East Asia, the prevalence ranged from 0% in Northern China to 97% in Southwestern China [51,86,87,88,89,90,91,92,93,94,95,96,97,98,99], and studies from Thailand in Southeast Asia averaged 13% prevalence [51,100,101,102]. No aflatoxin–albumin adducts were detected in Central and West Europe, although only 44 children and adults from France and 30 children and adults from Poland were included [51]. In North America, the prevalence ranged from 1% in the United States National Health and Nutrition Examination Survey (NHANES) [103] to 94% in two studies from Mexico [104,105]. Again, the within-country prevalence was highly variable; while the overall prevalence in the United States was 1% [103], it was 66% in a study from Texas [106]. In Central America, the prevalence was 100% in a study from Guatemala [107], while in South America, the prevalence was 21% from a study in Chile [108].

Restricting to studies that used blood samples collected in 2010 and later (Table 1), the continental regions with the highest prevalences were East Asia, with 100% prevalence in two studies from Guangxi, China [88,89], and Central America, with 100% prevalence in one study from Guatemala [107]. The prevalence was also quite high in West Africa, with 99% prevalence in two studies from the Gambia [56,57], and North America, with 92% prevalence in one study from Mexico [105]. The prevalence was only slightly lower in 8 studies from East Africa (87%) [67,69,70,71,72,73,76,77] and six studies from South Asia (80%) [79,81,82,83,84,85]. The only study from South America had a prevalence of 21% [108]. These investigations conducted within the last 15 years demonstrate that exposure to aflatoxin continues to be of concern and also that there are large gaps in our knowledge of the prevalence of aflatoxin exposure.

To date, studies of aflatoxin exposure in human biospecimens have been concentrated largely in Africa and Asia (Figure 2, Supplemental Table S1). Only a handful of studies from Europe and the Americas have estimated the prevalence of detectable aflatoxin–albumin adducts, and several of those were conducted 25 or more years ago. The paucity of data from South America is particularly ironic given that aflatoxins were originally discovered due to turkey poisoning from Brazilian groundnut meal that was highly contaminated with *Aspergillus flavus*, *Aspergillus parasiticus*, and *Aspergillus nomius* [15,109]. Despite this connection between South America and the discovery of aflatoxin, the potential impact on human disease of exposure to aflatoxin in Latin America has only been recognized recently [110,111,112]. The dearth of data from Latin America and the Caribbean has prevented public health recommendations due to the lack of evidence of the burden of cancers that might be attributable to aflatoxin in these regions [110]. A more thorough global survey of human exposure to aflatoxin is needed. New studies are also needed because broadscale changes in temperature and rainfall are shifting the geographic regions favorable for *Aspergillus* growth to the extent that regions that were previously at low risk for aflatoxin contamination, such as Europe, are experiencing increased crop contamination [113].

## 4. Aflatoxins and Human Disease

### 4.1. Non-Cancer Outcomes

Acute exposure to high levels of aflatoxin B_1_ can cause aflatoxicosis, a condition characterized by jaundice, fever, ascites, pedal edema, and vomiting [114]. There is evidence that aflatoxin can cause hepatotoxicity, although that outcome is more common in children than adults [12,114]. Children seem particularly susceptible to the effects of aflatoxin [14]. Aflatoxin has also been associated with increased risk of liver fibrosis and cirrhosis [115,116,117]. Animal data suggests that aflatoxin B_1_ can reduce fertility [12], and a study in Nigeria found that aflatoxin levels were higher in infertile men compared to fertile men.

Aflatoxin exposure has also been associated with malnutrition and adverse birth outcomes [12,118]. Several studies have found that children with kwashiorkor, a malnutrition disorder characterized by protein deficiency, have higher detection and levels of aflatoxins than children without [19,118]. Reverse causality or common causes such as diet or infection complicate causal inference, however. High aflatoxin B_1_–albumin adduct levels have also been associated with lower serum levels of vitamin A and vitamin C [119,120]. In addition, premature birth, perinatal death, low birthweight, and small for gestational age have all been associated with aflatoxin exposure [12,89,118,121,122]. Of these, low birthweight is one of the most consistently associated outcomes; evidence is more mixed for preterm birth, and there is low certainty for associations with child health outcomes [123]. Many studies do not adjust for maternal infections, socioeconomic factors, and diet, leading to concerns that observed associations could be due to confounding. Adverse birth effects might not be as pronounced with lower aflatoxin exposure levels, however [124].

Animal studies suggest that aflatoxin exposure leads to more frequent and severe infections, as well as longer duration of infection [11,125,126,127], likely driven by the immunotoxic effects of aflatoxin [128]. Studies in humans are more limited and more mixed. For example, one Gambian cohort of 391 children (323 with aflatoxin B_1_–albumin adduct measurement) found higher levels of aflatoxin B_1_–albumin adducts in children with *Plasmodium falciparum* parasitemia compared to those without and in children with seropositive for hepatitis B surface antigen (HBsAg) compared to those without [129], suggesting that aflatoxin B_1_ can increase risk of infection. In contrast, a United Arab Emirates study found no difference in the incidence of infection between 111 aflatoxin M_1_ seropositive and 55 aflatoxin M_1_ seronegative children [130], and aflatoxin levels were not associated with duration of fever from acute respiratory tract infections in a cohort of 115 children in the Philippines [131]. A better understanding of aflatoxin exposure and risk of infections in humans is needed.

### 4.2. Liver Cancer

Aflatoxins B_1_, G_1_, and M_1_ are the only mycotoxins that the International Agency for Research on Cancer [12] has classified as Group 1 carcinogens [12]. Extensive evidence indicates that aflatoxin causes liver cancer. Initial indications came from ecological studies conducted in sub-Saharan Africa and Asia from the 1960s through the 1980s, followed by case–control and prospective cohort studies [12].

**Figure 3:**
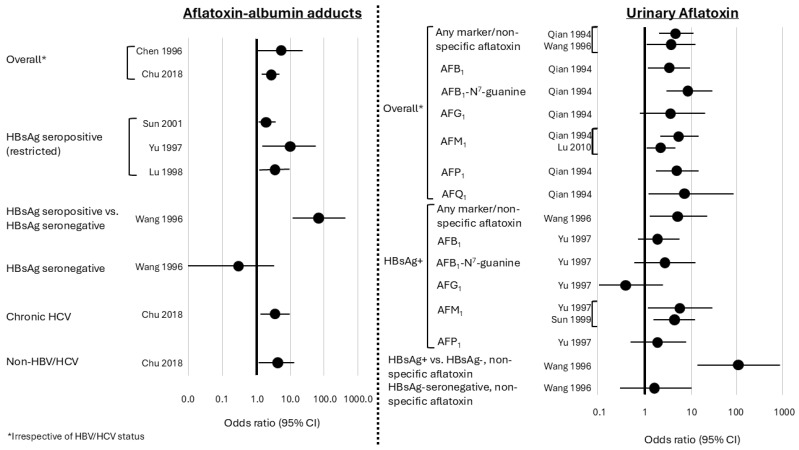
Odds ratios and 95% confidence intervals for the association between biospecimen-based aflatoxin exposure and liver cancer from case–control studies nested within cohorts [93,97,132,133,134,135,136,137,138].

Focusing on associations between biospecimen-based measurements of aflatoxins and HCC case–control studies nested within cohorts (Figure 3), which provide the most powerful observational evidence, Qian et al. measured urinary AFB-N7-gua (an aflatoxin-nucleic acid adduct), aflatoxin M_1_, aflatoxin P_1_ (another metabolite of aflatoxin B_1_), and aflatoxin B_1_ in a nested case–control study in Shanghai, China, of 50 HCC cases and 267 age-, time of sample collection-, and neighborhood-matched controls and found an HBsAg- and smoking-adjusted relative risk (RR) of 5.0 (95% CI: 2.1–11.8) for detection of any biomarker versus no biomarker detection and risk of HCC [132]. RRs for individual aflatoxin biomarkers ranged from 5.7 (95% CI 1.3–26.0) for aflatoxin B_1_ to 16.1 (95% CI 3.6–72.5) for aflatoxin M_1_. Furthermore, there was interaction between hepatitis B virus (HBV) and aflatoxin on the multiplicative scale, with an RR of 59.4 (95% CI: 16.6–212.0) for HCC among HBsAg-seropositive participants with any detectable aflatoxin biomarker compared to HBsAg-seronegative participants with no aflatoxin detection. Although the mechanisms are not fully understood, experimental data support the hypothesis that this interaction is biological, not merely statistical [127]. Additional nested case–control studies from China [93,133,134], and several nested case–control studies from Taiwan confirmed the strong association between aflatoxin and HCC [97,135,136,137,138,139]. For example, Wang et al. reported odds ratios ranging from 1.6 (95% CI: 0.4–5.5) for detectable versus nondetectable aflatoxin–albumin adducts to 111.9 (95% CI: 13.8–905.0) for high levels of urinary aflatoxin plus HBsAg seropositivity versus low levels of urinary aflatoxin among HBsAg seronegative individuals [137]. Nested case–control studies from China provided additional evidence of strong associations of aflatoxin B_1_–albumin adducts and urinary aflatoxin M1 with HCC/liver cancer [93,133,134]. Together, these findings provide strong support for IARC’s classification of aflatoxins B_1_, G_1_, and M_1_ as Group 1 carcinogens [12].

### 4.3. Gallbladder Cancer

In addition to HCC, there is increasing evidence that aflatoxin B_1_ might cause gallbladder cancer (GBC). Animal studies have shown that aflatoxin exposure increases biliary proliferation [140,141,142,143,144], and necropsy after an aflatoxicosis outbreak identified extensive proliferation in human bile duct samples as well [145]. The biliary tract might be particularly vulnerable to the toxic effects of aflatoxin since aflatoxin is excreted into bile and can become highly concentrated in the biliary tree [146,147]. Furthermore, an aflatoxin feeding study in rhesus and cynomolgus monkeys demonstrated that primates exposed to aflatoxin can develop biliary tract cancers, including GBC [148]. Adenosine triphosphate-binding cassette (ABC) transporter pumps move cholesterol and xenobiotics like aflatoxin B_1_ from the liver into the biliary tract via bile [149]. Given that genetic variants in ABC transporter genes have been shown to confer risk of both gallstones (the strongest risk factor for GBC) [150,151,152,153,154] and GBC [155,156] and that the risks associated with these variants might be particularly strong in distinct population groups [155], it seems reasonable to hypothesize that interactions between these genes and aflatoxin B_1_ could increase risk of GBC while decreasing risk of HCC depending on which variants are prevalent in a given population.

There is both indirect and direct evidence for an association between aflatoxin B_1_ and risk of GBC in humans. The first indirect evidence came from an occupational study in Denmark, which found that workers who processed feed for livestock were more likely to develop GBC and extrahepatic bile duct cancer (EHBDC) than the general population (standardized proportional incidence ratio [SPIR]: 219, 95% CI: 89–455) [157]. The authors hypothesized this increased risk was due to high levels of aflatoxin observed in the dust of feed processing companies. Additional indirect evidence came from a case–control study of 114 GBC cases and 114 matched hospital controls in Santiago, Chile, which found that GBC cases were more likely to consume red chili pepper than controls (OR: 2.9, 95% CI: 1.6–5.2) [158]; red chili peppers from this region were later shown to be contaminated with aflatoxin B_1_ and aflatoxin G_1_ [159].

A Chilean case–control study provided the first direct evaluation of aflatoxin and GBC in humans, finding ORs of 9.4 (95% CI: 2.8–37.2) and 13.2 (95% CI: 4.3–47.9) for detectable versus circulating aflatoxin B_1–_albumin adducts in GBC cases compared to gallstone and community-based controls, respectively [108] (Table 2). This study was followed by a population-based case–control study from Shanghai, China, which found an OR of 2.7 (95% CI: 1.7–4.3) for detectable versus undetectable aflatoxin B_1_ and GBC compared to gallstone controls [95]. Aflatoxin B_1_ detection versus non-detection was strongly associated with GBC compared to a combined group of gallstone patients and normal controls in a hospital-based case–control study from Chandigarh, India (OR: 6.8, 95% CI: 1.3–35.7) for [81]. Further support came from another hospital-based case–control study from Jaipur, India, where the OR for aflatoxin B_1_ detection versus non-detection was 4.1 (95% CI: 1.7–9.8) for GBC compared to gallstone controls, and 16.8 (95% CI: 4.0–70.2) for GBC compared to healthy liver donors [82]. Finally, a nested case–control study from Shanghai, China, provided the first evidence of temporality (i.e., that exposure preceded outcome), with an OR of 2.0 (1.0–3.9) for 84 GBC cases that developed over more than 30 years of follow-up in a cohort of over 18,000 men [96]. There is some evidence of dose response in the association between aflatoxin and GBC as well (e.g., OR 1.2 [95% CI: 0.3–5.8] for quartile 2 versus 1 and 7.6 [95% CI: 2.0–28.8] for quartile 4 versus 1 in Koshiol et al. [95] and OR 10.6 [95% CI: 0.6–194.5] for quartile 2 versus 1 and 36.6 [95% CI: 2.0–653.3] for quartile 4 versus 1 in Yadav et al. [81]. For estimates that are based on comparison to patients with gallstones, it is important to note that gallstones are a strong risk factor for GBC, which effects interpretation of effect sizes. The magnitudes of the estimates for GBC versus gallstones were lower than those for GBC versus non-gallstone controls in case–control studies, but higher than the nested case–control estimate.

Finally, a molecular profiling study of GBC cases from China and Chile detected the single base substitution mutational signature that has been linked to aflatoxin exposure (COSMIC signature 24) [160,161] in 39 out of 92 cases (42.4%), providing additional molecular support for a role of aflatoxin in gallbladder carcinogenesis [162]. Based on circulating aflatoxin B_1_–albumin adduct data, Koshiol et al. estimated the population-attributable fraction for GBC related to aflatoxin to be 20% (95% CI, 15–25%) in the population-based case–control study from Shanghai, China, and 52% (95% CI, 38–63%) in the Chilean case–control study [95]. Although the number of studies is small and most are from regions with a high risk of GBC, limiting generalizability, taken together, the findings suggest a substantial, but not exclusive, etiologic role for aflatoxin in certain populations.

### 4.4. Other Cancers

While other cancers have been proposed to be associated with aflatoxin, evidence is more limited for those anatomic sites. Intrahepatic and extrahepatic bile duct cancers (IHBDC, EHBDC) are good candidates given the impact of aflatoxin on the biliary tract described above and animal data indicating that aflatoxin can cause bile duct cancer in ducks [43], glutathione S-transferase A3 knockout mice [163], and primates [148]. Studies of aflatoxin and primary liver cancer, which is predominated by HCC but also includes IHBDC, did not specify if the risk of IHBDC was increased or if the increased risk was due entirely to HCC [44,133]. One study in Thailand including 20 intrahepatic cholangiocarcinoma/hospital control pairs tested for circulating aflatoxin–albumin adducts and found no association with aflatoxin (OR: 1.0, 95% CI: 0.1 = 16.0) [100]. The association of circulating aflatoxin B_1_–albumin adduct levels with EHBDC has not been directly assessed in humans. Studies of aflatoxin B_1_ DNA adducts and mutational signatures have produced mixed results. Gramantieri et al. [164] used immunohistochemistry to assess aflatoxin B_1_ DNA adducts in tumor tissue samples 131 HCC and cholangiocarcinoma cases. They found aflatoxin B_1_ DNA adducts in 25 (19%) of the HCC cases, but none of the cholangiocarcinoma cases, suggesting no association between aflatoxin and bile duct cancers. Conversely, Villar et al. measured plasma R249S-mutated DNA, which has been used extensively as a biomarker for aflatoxin exposure [43], and identified R249S-mutated DNA in 22% of 45 cholangiocarcinoma patients, similar to the 21% of 36 HCC patients with cirrhosis (though lower than the 44% of 50 HCC patients without cirrhosis) and notably higher than the 12% in 56 patients with chronic liver disease and 3% of 133 individuals undergoing an annual health exam without clinical evidence of liver disease [165]. Molecular characterization studies have not reported the aflatoxin-related single base substitution mutational signature 24 in bile duct cancers [166,167,168], although these studies have been based largely on cases from Japan, where there is little aflatoxin exposure [12,169]. In contrast, a Chinese study of 204 IHBDC patients identified a C > A mutational signature they termed “signature F” and found that this signature was highly correlated with COSMIC signature 24 [170]. Studies that directly measure aflatoxin exposure and assess risk of IHBDC and EHBDC are needed to clarify the association of aflatoxin with bile duct cancers.

Since aflatoxin can enter the body through inhalation, it has also been proposed to increase the risk of lung cancer. Animal and in vitro studies suggest that aflatoxin can cause cancer in the lung [12,171,172,173]. Epidemiologic findings have been mixed, however. Hayes et al. compared mortality in 71 oil press workers who worked in an aflatoxin-exposed area for at least 2 years to mortality in 67 unexposed workers in the Netherlands and identified an elevated standardized mortality ratio (SMR) for respiratory cancers (International Classification of Diseases, 9th edition codes 160–163) in exposed workers (SMR: 2.5, 95% CI: 1.0–5.0) [12,174]. In contrast, another occupational study from Denmark found fewer lung cancer cases in exposed workers than expected (SPIR for expected to observed: 74, 95% CI: 58–95) [157]. Experimental studies have also implicated aflatoxin in the development of gastrointestinal, kidney, and skin cancers [173,175], but epidemiologic data are lacking. Current epidemiologic data are insufficient to draw firm conclusions for cholangiocarcinoma, lung, kidney, and gastrointestinal cancers. Any inferences remain speculative.

## 5. Biologic Mechanisms for Carcinogenesis

Aflatoxin B_1_ is a genotoxic agent. After absorption through the duodenum, it is metabolized in the human liver by the cytochrome P450 (CYP450) oxidase family, primarily CYP3A4 and CYP1A2, into aflatoxin B_1_-exo 8,9-epoxide (AFBO), which has endo-8,9-epoxide and exo-8,9-epoxide isomers [11,118,176]. AFBO is highly electrophilic, allowing it to covalently bond with the N7 position on guanine base in DNA. The resulting DNA adducts cause persistent DNA damage that can be missed by DNA repair, leading to DNA mutations.

In addition to mutagenic DNA adducts, aflatoxin B_1_ metabolism creates oxidative stress, mitochondrial dysfunction, and inflammation [11,176,177,178]. Depending on the dose and timing of aflatoxin exposure, aflatoxins can have immunosuppressive or immunostimulatory effects [11,118,128]. Aflatoxin B_1_ can also cause epigenetic changes impacting cell cycle regulation, apoptosis, oxidative stress, and malignant transformation [177]. In addition, animal studies have demonstrated that aflatoxin can induce ferroptosis, a kind of regulated cell death characterized by iron-dependent lipid peroxidation accumulation that could contribute to carcinogenesis through liver damage and immune cell dysfunction [179,180,181,182,183,184].

The carcinogenic effects of aflatoxin B_1_ can also be amplified by other factors. For example, several epidemiologic studies have found that the joint effects of aflatoxin B_1_ and HBV infection are greater than would be expected by simply multiplying the individual effects [132,137,185], which has been supported by a number of in vitro and in vivo studies [127]. Less work has been done to evaluate potential synergy between hepatitis C virus (HCV) and aflatoxin, but a Taiwanese study found that detectable versus nondetectable aflatoxin B_1_–albumin adducts were associated with advanced liver disease in anti-HCV-positive participants (OR: 2.1, 95% CI: 1.1–4.0) [186]. Another study found that HCV transgenic mice demonstrated more pronounced effects of aflatoxin B_1_-induced inflammation and altered lipid metabolism than aflatoxin B_1_-treated wild type mice [187]. While several mechanisms have been proposed [188], the biological processes involved in the synergy between HBV, and possibly HCV, remain unclear [11].

Mycotoxins can also co-occur and might act synergistically. For example, aflatoxins and fumonisins often exist simultaneously in corn [12,189]. Based on animal data, IARC classified fumonisin B_1_ as “possibly carcinogenic to humans” (group 2B) [12]. Experimental data suggest that aflatoxin B_1_ and fumonisin B_1_ could have a synergistic effect on apoptosis and increase hepatocarcinogenicity [190]. Epidemiologic data are lacking.

## 6. Measures to Minimize Aflatoxin Exposure

**Figure 4:**
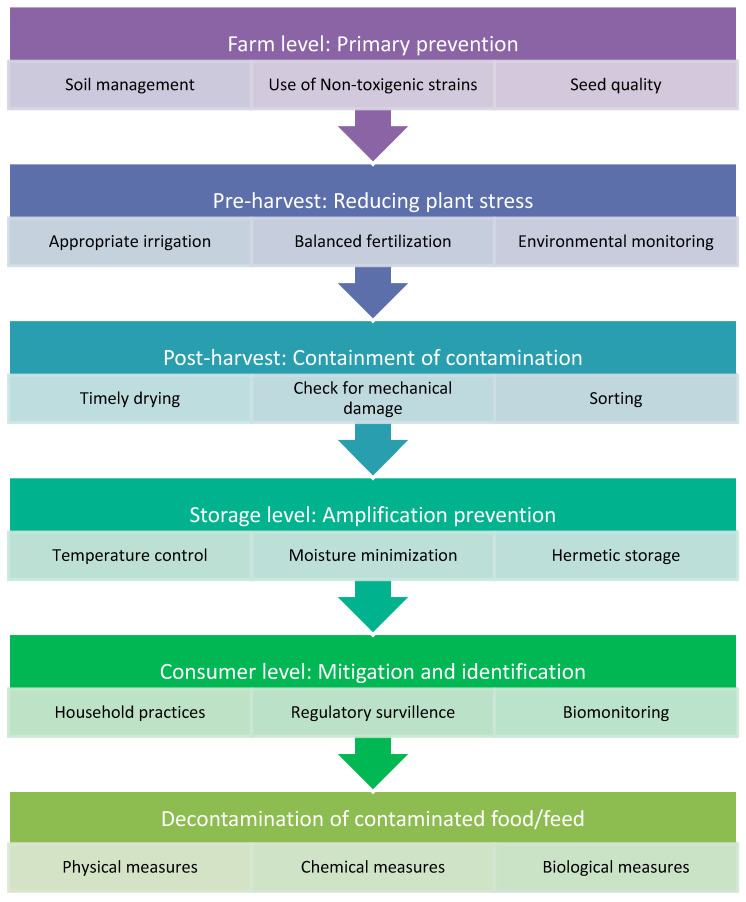
Multi-level interventions for aflatoxin prevention, mitigation, and control along the food supply. Effective aflatoxin control requires a stepwise framework of strategies to reduce aflatoxin contamination from production to consumption. At the farm level, primary prevention includes soil management, use of non-toxigenic *Aspergillus* strains, and seed quality improvement. Pre-harvest practices focus on reducing plant stress through adequate irrigation, balanced fertilization, and environmental monitoring. Post-harvest measures aim to limit contamination by timely drying, assessment of mechanical damage, and sorting. Storage-level interventions prevent aflatoxin amplification by controlling temperature and moisture and applying hermetic storage. At the consumer level, mitigation and identification involve household practices, regulatory surveillance, and biomonitoring. Finally, decontamination strategies encompass physical, chemical, and biological approaches to reduce aflatoxin levels in food and feed.

Unlike many food contaminants, aflatoxin contamination is not confined to a single point in the food chain but represents an accumulating risk that begins in the soil and may intensify during cultivation, harvesting, storage, processing, and final consumption. Consequently, effective control cannot rely on a single intervention but requires a continuum of preventive and mitigative strategies operating at multiple levels (Figure 4). In resource-constrained environments, several interventions have demonstrated good results in reducing aflatoxin levels. Use of atoxigenic *Aspergillus* biocontrol strains, high-quality seed, and soil management reduces baseline contamination at the farmer level. Stress-induced aflatoxin production is reduced by pre-harvest techniques, such as irrigation and pest control. Further contamination is successfully prevented by post-harvest methods such as rapid drying, careful handling, and easy sorting of damaged kernels. Hermetic technologies and moisture control prevent exponential accumulation of aflatoxin during storage. Together, these strategies offer the most realistic and scientifically proven methods for reducing aflatoxin in settings with limited resources.

### 6.1. Farm-Level Interventions: Primary Prevention at the Source

The foundation of aflatoxin control lies at the farm level, where contamination begins long before crops enter the food system. Soil acts as the principal ecological reservoir for *Aspergillus* species, particularly *Aspergillus flavus* and *Aspergillus parasiticus*. Fungal density and composition are influenced by soil structure, organic matter content, cropping patterns, and historical land use [7]. Soil management remains a critical but underemphasized determinant of aflatoxin risk. Soils with poor organic matter, low microbial diversity, and high compaction tend to favor *Aspergillus* survival. Practices such as crop rotation with non-host crops, incorporation of organic residues, conservation tillage, and maintenance of soil moisture can reduce fungal inoculum and competitiveness. In contrast, continuous monocropping enriches toxigenic strains and increases the probability of kernel colonization in subsequent seasons [191,192].

Seed quality represents another pivotal control point. Use of high-quality seeds reduces the introduction of fungal inoculum. Seeds with mechanical damage, microcracks, or latent fungal infection are more susceptible to colonization during growth and storage [7]. Genetic resistance to aflatoxin contamination is complex and polygenic, but cultivars with intact seed coats, tight husk coverage, antifungal proteins, and rapid kernel drying demonstrate lower susceptibility. One of the most significant advances in farm-level primary prevention is the use of atoxigenic strains of *Aspergillus flavus* as biological control agents. These strains competitively displace toxigenic strains in the soil and on developing crops. Field trials across Africa, the United States, and parts of Asia have consistently demonstrated reductions in aflatoxin contamination ranging from 70% to over 95% when atoxigenic strains are properly applied [193]. The effectiveness of this approach lies in ecological replacement rather than eradication, making it sustainable across multiple seasons.

### 6.2. Pre-Harvest Level: Reducing Plant Stress and Fungal Entry

While primary prevention reduces baseline risk, pre-harvest factors often determine whether aflatoxin biosynthesis is initiated during crop development [194]. Aflatoxin production is strongly influenced by plant stress and biotic damage. Warm temperatures and drought favor the persistence and dominance of toxigenic strains. Water stress compromises plant defense mechanisms, disrupts kernel integrity, and enhances fungal colonization [195].

Adequate and timely irrigation during critical growth periods is one of the most effective pre-harvest interventions [196]. Balanced fertilization also plays a role, as excessive nitrogen can promote lush vegetative growth without proportional kernel protection, increasing susceptibility to fungal invasion. Insects create physical breaches in kernels, providing direct access for *Aspergillus* spores and facilitating internal colonization where aflatoxins are less amenable to removal. Integrated pest management strategies such as biological control, crop sanitation, and judicious insecticide use significantly reduce risk [197].

The role of fungicides in aflatoxin control is limited and indirect. *Aspergillus* species are poor targets for conventional fungicides. Fungicides may contribute by reducing plant stress from other fungal diseases, thereby indirectly lowering aflatoxin risk. Environmental monitoring during high-risk growth stages can help predict contamination and inform targeted interventions [198].

### 6.3. Post-Harvest Level: Containment of Contamination

The post-harvest phase represents a critical amplification point where modest field contamination can escalate into severe toxin burdens if handling practices are inadequate. Once crops are harvested, the protective physiological defenses of the plant cease, and kernels become highly vulnerable to fungal proliferation if moisture and temperature conditions are favorable. Delayed drying is one of the most important determinants of increases in aflatoxin contamination post-harvest. Moisture levels above safe thresholds allow rapid fungal growth and toxin production within hours to days. Crops should therefore be harvested at physiological maturity and dried as rapidly as possible, ideally within 24 to 48 h, to moisture contents below levels that support fungal activity [7,199]. Traditional sun drying is dependent on weather conditions and may be insufficient in humid environments unless supplemented with raised platforms, frequent turning, and protection from rewetting.

Mechanical damage during harvesting, shelling, and transport further exacerbates risk by exposing internal tissues and facilitating fungal penetration [200]. Gentle handling, proper adjustment of harvesting equipment, and avoidance of excessive shelling force reduce kernel breakage and subsequent contamination. Sorting is one of the most powerful and cost-effective post-harvest interventions. Aflatoxin is often concentrated in a small fraction of visibly damaged, moldy, or discolored kernels. Manual or mechanical removal of these fractions can reduce overall aflatoxin levels by 50–80% or more, even though a proportion of contaminated kernels may appear visually normal. Sorting does not eliminate aflatoxins but substantially lowers exposure and serves as a critical containment measure before storage or processing [201].

### 6.4. Storage Level: Preventing Exponential Amplification

Storage conditions determine whether aflatoxin levels remain stable or increase exponentially over time. Even crops that meet regulatory limits at harvest may exceed permissible thresholds after weeks or months of improper storage. Moisture content is the single most important factor governing fungal growth during storage. For most cereals, moisture levels should be maintained below 14%, while oilseeds and nuts require even lower levels, typically below 7–8%. Relative humidity above 70% promotes moisture migration within storage structures, leading to localized “hot spots” of fungal activity. Temperature interacts with moisture to influence aflatoxin risk. While *Aspergillus* species can survive across a wide temperature range, toxin production is optimized at warm temperatures. Storage temperatures below 25 °C significantly reduce fungal metabolism, though such conditions may be difficult to achieve in tropical settings without structural modifications [7,199,202].

Hermetic storage has emerged as a highly effective intervention, particularly for smallholder systems. By creating an airtight environment, it deprives fungi and insects of oxygen and suppresses growth and prevent aflatoxin biosynthesis even when initial contamination is present [203]. Modified atmosphere storage using carbon dioxide or nitrogen further enhances this effect and is increasingly adopted in commercial settings. Proper packaging materials, moisture barriers, and regular monitoring for condensation, temperature spikes, and structural integrity are essential components of storage-level control [204].

### 6.5. Consumer-Level Mitigation and Identification of Contamination

At the consumer level, aflatoxin control shifts from prevention to exposure reduction, as contamination may already be present in purchased food products. Identification of aflatoxin contamination at this stage is inherently challenging because aflatoxins are colorless, odorless, and tasteless at low concentrations. Nevertheless, certain warning signs can raise suspicion, including visible mold growth, musty or bitter odors, discoloration, and a history of poor storage conditions. However, the absence of these signs does not guarantee safety.

Household-level practices such as washing, soaking, dehulling, roasting, and cooking can reduce surface contamination and partially degrade aflatoxins, but they are insufficient to eliminate internally bound toxins. Thermal processing reduces aflatoxin levels to only a limited extent because aflatoxins are relatively heat stable under conventional cooking conditions. Consequently, consumer-level interventions should be viewed as risk-reduction measures rather than definitive solutions.

Regulatory surveillance and food testing remain essential for consumer protection. Rapid test kits, immunoassays, and laboratory-based chromatographic methods provide varying levels of sensitivity and specificity. Public awareness, labeling, and enforcement mechanisms are critical to prevent contaminated food from entering formal markets, particularly in regions where informal food systems dominate.

### 6.6. Aflatoxin Decontamination Strategies

The prevention of mycotoxin contamination prior to harvest or during post-harvest storage is not always feasible, making decontamination of contaminated food and feed materials necessary to reduce toxic and carcinogenic risks. The overarching objectives of decontamination include removal of contaminated material, chemical or structural inactivation of the toxin, or prevention of absorption in the host. Various detoxification strategies play a crucial role in mitigating exposure to mycotoxins. Detoxification can be achieved either by removal/elimination of contaminated commodities or by inactivation of the toxins through physical, chemical, or biological methods.

An effective decontamination process for mycotoxins must fulfill several essential criteria to ensure both safety and practicality. Firstly, it should efficiently destroy, inactivate, or remove the mycotoxins present in contaminated materials. Equally important is that the process must not generate any toxic, carcinogenic, or mutagenic residues in the treated products or in foods derived from animals consuming the decontaminated feed. Additionally, the procedure should maintain the desirable physical and sensory qualities of the product, such as appearance, taste, and texture. It should also eliminate fungal spores and mycelium to prevent the reformation of mycotoxins under favorable environmental conditions. Finally, for wide-scale adoption, the method must be both technically feasible and economically viable, ensuring that it can be applied efficiently without imposing excessive costs [205,206]. Aflatoxin decontamination approaches are broadly categorized into physical, chemical, and biological methods.

#### 6.6.1. Physical Methods

Physical segregation of contaminated crops is a practical and important method for reducing mycotoxin levels, especially where chemical treatments are restricted. Traditional physical interventions such as manual or mechanical sorting, density segregation, dehulling, and milling are accepted for food processing to reduce aflatoxin levels and are compatible with regulatory standards. Chemical treatments and biological methods are more commonly used in animal feed applications, as regulatory approval for direct use in food intended for human consumption is limited and concerns remain about residues and product quality. Novel technologies such as cold atmospheric plasma and high-dose gamma irradiation show promising efficacy in experimental and pilot studies but are currently largely at the research or industrial scale stage and lack widespread regulatory approval and commercial implementation for food use. These techniques also require careful assessment of degradation products, impacts on food quality, and feasibility for large scale integration into food processing systems to ensure they meet safety and regulatory requirements

(a) Sorting: Most mycotoxin contamination is concentrated in a small fraction of seeds or kernels. Sorting damaged, discolored, or visibly moldy crops can remove a significant portion of mycotoxins. This low-cost intervention can achieve substantial toxin reduction, particularly in small-scale settings [207]. Methods include manual, mechanical, and electronic sorting:
  - Manual selection: Damaged kernels are identified based on size, shape, color, and visible mold.
  - Fluorescence sorting: Contaminated maize, cottonseed, and dried figs can be detected under UV light (365 nm) due to bright greenish-yellow fluorescence correlated with aflatoxin presence [208].
  - Electronic sorting: Though effective, large-scale industrial application is limited due to cost. Combined electronic and manual sorting is used in the peanut industry to reduce aflatoxin levels [209].
(b) Density Segregation: Buoyant kernels in water or saturated salt solutions can be removed. Specific gravity tables can remove low-density “tombstone” kernels, reducing mycotoxins in wheat by 68–85% [210,211].
(c) Washing: Washing grains can significantly reduce mycotoxin levels. Repeated washing of barley or maize with water reduces mycotoxins by 65–69% [210].
(d) Milling: Removal of grain components during milling can lower mycotoxin content, Fumonisins are distributed unevenly in maize milling fractions, being lower in flaking grits and higher in germ and bran.
(e) Dehulling and polishing: Aflatoxins are predominantly localized in the outer layers of grains. Removal of the hull or bran through dehulling or polishing significantly lowers toxin content, albeit at the expense of some nutritional components.
(f) Heat Treatment: Most mycotoxins are relatively heat-stable under conventional food processing (80–121 °C). Thermal degradation depends on toxin type, moisture, pH, heating time, and temperature. Aflatoxins decompose at 237–306 °C; increased moisture enhances degradation. Roasting peanuts at 150 °C for 30–90 min reduces aflatoxins by 30–60%. Microwave and extrusion cooking have also been effective in degrading mycotoxins while maintaining food quality [212].
(g) Ionizing Radiation: Gamma and X-rays can degrade mycotoxins, a process termed “cold pasteurization” [213]. γ-radiation reduces aflatoxin B_1_ in peanuts by 75–100% at 1–10 kGy. UV radiation (222–362 nm) can degrade aflatoxin M_1_ in milk, aflatoxin B_1_ in dried figs, and peanut oil. Solar energy also contributes to mycotoxin degradation, with up to 70% of aflatoxin B_1_ in coconut oil destroyed under sunlight [214].
(h) Cold atmospheric plasma: Cold atmospheric plasma (CAP) is an emerging non-thermal technology that degrades aflatoxin through reactive oxygen and nitrogen species generated at atmospheric pressure. CAP disrupts fungal cell walls and breaks key chemical bonds within the aflatoxin molecule, leading to detoxification without significant nutrient loss. Although highly effective and residue free, CAP currently remains limited to industrial scale applications due to cost and infrastructure requirements. Nevertheless, it represents one of the most promising future technologies for large-scale aflatoxin control [215].
(i) Nixtamalization: Nixtamalization is an alkaline cooking process used traditionally in maize tortilla preparation. Nixtamalization is one of the few household cooking methods proven to significantly reduce aflatoxin levels. The alkaline conditions hydrolyze the lactone ring of aflatoxin B_1_, reducing bioavailability and genotoxicity [216].
(j) Extraction with Solvents: Several solvents can extract mycotoxins from contaminated food and oilseeds, including ethanol, acetone, isopropanol, hexane, and methanol–water mixtures. While effective in removing aflatoxins without toxic byproducts, large-scale applications are limited by cost and disposal issues. For example, 80% isopropanol can remove aflatoxins from cottonseed and peanut meal but also extracts 8–9% of solids [217].

#### 6.6.2. Chemical Methods

Chemical detoxification involves the use of acids, bases, oxidizing agents, reducing agents, chlorinating agents, and other reagents to inactivate or remove mycotoxins from contaminated foods and feeds [218,219]. Regulatory approvals for chemical decontamination are currently limited. Therefore, residues and product quality must be carefully evaluated before application to food for human consumption.

(a) Acids: Strong acids such as hydrochloric acid (HCl) and sulfuric acid (H_2_SO_4_) can convert aflatoxins aflatoxin B_1_ and aflatoxin G_1_ into less toxic hemiacetal forms aflatoxin B_2a_ and aflatoxin G_2a_ through hydration reactions. These treatments can significantly reduce the toxicity of aflatoxins when applied under controlled conditions.
(b) Bases (Ammoniation and Alkalis): Ammoniation is widely employed for the detoxification of aflatoxin-contaminated peanuts, cottonseed, and maize. High-pressure and high-temperature ammoniation treatments can destroy more than 99% of aflatoxin B_1_. Other alkaline treatments, such as sodium hydroxide, have also been shown to reduce aflatoxin levels effectively, though their efficacy is slightly lower than that of ammoniation [205].
(c) Oxidizing Agents: Oxidizing agents, including ozone (O_3_) and hydrogen peroxide (H_2_O_2_), are used to degrade mycotoxins. Ozone reacts with the C=C double bonds in aflatoxins such as aflatoxin B_1_, aflatoxin G_1_, and aflatoxin M_1_, leading to rapid degradation. Hydrogen peroxide has also been shown to be effective against aflatoxins, zearalenone (ZEN), and deoxynivalenol [220].
(d) Reducing Agents: Reducing agents, such as sodium bisulfite, detoxify aflatoxins by forming sulfonate derivatives, thereby reducing their mutagenic potential. The use of sorbic acid in combination with heat or UV treatments has been shown to further enhance the degradation of aflatoxins [221].
(e) Chlorinating Agents: Chlorine and sodium hypochlorite are effective in degrading aflatoxins and patulin in food products. They are commonly used in the food industry as sanitizing agents and for decontamination of raw materials [222].
(f) Miscellaneous Reagents: Other chemicals, including formaldehyde, potassium permanganate, and sodium borate, have demonstrated detoxification potential. However, their use is limited due to safety concerns and the possibility of producing toxic residues, restricting their application in food and feed [7].

#### 6.6.3. Biological Methods

Biological detoxification is gaining increasing attention due to its safety, cost-effectiveness, and minimal impact on food quality. Various strategies involve microbial degradation, fermentation, inhibition of absorption in the gastrointestinal tract, competitive exclusion, and enzymatic degradation. It is important to note that the efficacy of microbial degradation and adsorbent binders is strain- and matrix-dependent, and in vivo benefits may not always reflect in vitro binding results

(a) Microbial degradation: Certain fungi and bacteria can directly degrade mycotoxins. Fungi such as Trichoderma, Phoma, Rhizopus, and Alternaria can degrade aflatoxin B_1_ by 65–99% within five days. Bacteria such as Acinetobacter calcoaceticus are capable of completely degrading mycotoxins in liquid media, highlighting the potential of microbial systems for targeted detoxification [223].
(b) Fermentation: Fermentation processes with yeasts and bacteria can reduce mycotoxin levels in foods and feeds. Fermentation with Candida intermedia decreases F2 toxin (zearalenone) activity by tenfold [224]. Ethanol fermentation of maize, beer, apple juice, and barley effectively reduces mycotoxins. Lactic acid bacteria and bifidobacteria can degrade aflatoxin B_1_ and aflatoxin M_1_ during milk fermentation, thereby improving food safety while maintaining nutritional quality.
(c) Inhibition of absorption in the gastrointestinal tract: Biological binders and probiotics can reduce mycotoxin bioavailability in livestock. Non-nutritive adsorbents such as activated carbon, hydrated sodium calcium aluminosilicate (HSCAS), zeolites, bentonites, and esterified glucomannan prevent systemic absorption of mycotoxins [225]. Lactic acid bacteria and bifidobacteria bind aflatoxins through cell wall interactions, hydrophobic binding, and polysaccharide–peptidoglycan complexes. Binding efficiency varies among bacterial strains; for example, Lacticaseibacillus rhamnosus GG can reduce aflatoxin B_1_ bioavailability by up to 80%, ultimately lowering aflatoxin M_1_ excretion in milk [226].
(d) Competitive exclusion: As described above, application of non-aflatoxigenic Aspergillus strains in agricultural fields can competitively suppress toxigenic strains, reducing aflatoxin production at the source. This approach has been successfully implemented in large-scale crop interventions to prevent contamination before harvest.
(e) Preventing adsorption in livestock: In animal production, feed additives such as clay-based binders, aluminosilicates, activated charcoal, Saccharomyces cerevisiae, and lactic acid bacteria are incorporated to reduce intestinal absorption of aflatoxin B_1_. These biological binders limit systemic absorption and aflatoxin M_1_ excretion in milk, achieving reductions of 40–80% in dairy systems. The primary mechanism involves binding via cell wall components such as β-glucans and polysaccharides, which sequester toxins in the gastrointestinal tract [218].
(f) Enzymatic degradation: Microbial enzymes such as laccases and peroxidases can structurally modify aflatoxin B_1_, reducing its ability to form DNA adducts and decreasing carcinogenic potential. Recent advances in computational biology and artificial intelligence have enabled the design of optimized enzymes and predictive modeling of enzyme–toxin interactions, representing a move toward precision detoxification strategies.

### 6.7. Developments in Biomonitoring

Biomonitoring is a key component of an integrative program to minimize aflatoxin exposure. A number of emerging technologies offer great promise in this area. For example, electrochemical biosensors (impedimetric, amperometric, and voltametric) can sensitively and rapidly detect the presence of aflatoxins through use of antibodies (e.g., anti-aflatoxin B_1_) or aptamers (DNA or RNA sequences) that bind to an aflatoxin target, such as aflatoxin B_1_ [227,228]. Recent advances have allowed integration of these devices with smartphones and other mobile devices. While widespread use has been limited by the lack of standardized, non-customized biosensors and the need for an experienced operator, newer, polymer-based devices may help alleviate these challenges while providing greater sensitivity [228].

Artificial intelligence (AI) through machine learning is another important avenue of advancement. AI algorithms have been developed to distinguish between contaminated and uncontaminated kernels and to quantify contamination [227]. In addition, AI models can predict areas of contamination using environmental and agronomic data [227,228]. They can also be combined with biosensors to improve detection of aflatoxins, and they can provide support for real-time decisions based on detected levels of contamination [227]. However, many electrochemical biosensors and AI-based classification systems have been validated mainly in experimental or pilot settings; large scale implementation is still emerging.

New tests to detect aflatoxin exposure in humans would also assist biomonitoring efforts. The R249S mutation in TP53 has long been considered a valuable tissue-based marker of exposure to aflatoxin B_1_ [43], but some studies suggest that this particular mutation might reflect interaction with HBV and therefore might not be a universal indication of exposure to aflatoxin [165,229,230,231,232]. More recently, the COSMIC signature 24 single base substitution mutational signature, which includes high frequencies of G-C to T-A transversions, has been linked to aflatoxin exposure [43,160,161]. Blood-based assays using dried blood spots, dried serum spots, and volumetric tip microsampling have also been developed for multiple mycotoxins [233]. These assays could potentially have an important role in human biomonitoring, although they are currently used for research rather than operational surveillance. Most studies have been performed in small populations, the sensitivity can be low for some mycotoxins in multimycotoxin assays, and volumetric tip microsampling has a high cost relative to dried blood spot sampling. Thus, more work is needed to improve the sensitivity of these assays and validate their use in real-world settings [233].

## 7. International Regulations and Standards for Aflatoxin Control

The recognition of aflatoxins as serious threats to human and animal health in the early 1960s led to the introduction of regulatory limits for mycotoxins in food and feed across the world. The first aflatoxin limits were established in the late 1960s, and by 2003 nearly 100 countries had adopted mycotoxin regulations [234]. However, regulatory coverage remains uneven, with many developing and African countries lacking enforceable standards, largely because strict limits could worsen food shortages and increase costs. Globally, regulations focus mainly on aflatoxin B_1_, total aflatoxins, and aflatoxin M_1_ [235]. The Codex Alimentarius sets maximum limits of 15 µg/kg for total aflatoxins in nuts for further processing, 10 µg/kg for nuts and dried figs for direct consumption, and 0.5 µg/kg for aflatoxin M_1_ in milk, although national limits vary widely depending on food type and trade policies [23]. The European Union enforces strict aflatoxin standards through the Commission Regulation (EU) 2023/915 of 25 April 2023 on maximum levels for certain contaminants in food, which repeals Regulation [5] No 1881/2006. Under this framework, the maximum permitted limits range from 2–12 µg/kg for aflatoxin B_1_, 4–15 µg/kg for total aflatoxins, and 0.025–0.05 µg/kg for aflatoxin M_1_ in milk, which is 10–20 times lower than Codex limits [236]. In comparison, higher maximum limits are permitted in many countries, including 30 µg/kg for aflatoxin B_1_ in India, 20 µg/kg in the Philippines, and 15–20 µg/kg in Indonesia, with variable ranges across China, Japan, Korea, the United States, Brazil, and several African nations [23,237]. Most countries that regulate aflatoxin M_1_ in milk adopt a limit of 0.5 µg/kg, consistent with Codex. Aflatoxins can also contaminate water, but standards for aflatoxin levels in water have not yet been developed [228]. The lack of regulatory limits for aflatoxins in water likely reflects the limited evidence for water as a significant exposure route relative to food. Low-level contamination has been reported; however, its health impact remains uncertain. Overall, aflatoxin regulations are central to exposure prevention, but global disparities in limits reflect differences in risk assessment, food security concerns, and regulatory capacity (Table 3).

## 8. Conclusions

Of all the mycotoxins, aflatoxins have the biggest known impact on human health. Acute aflatoxin B_1_ exposure can cause aflatoxicosis and death. Aflatoxin exposure has also been associated with infertility, adverse birth outcomes, malnutrition, and more frequent and severe infections. Chronic exposure to aflatoxin B_1_ can cause liver cancer, and mounting evidence suggests it might cause gallbladder cancer. In this review, we noted several key research gaps, including (1) a lack of contemporary exposure data in Latin America and many high-income regions, (2) limited evidence for non-HCC cancers and for infection-related outcomes, and (3) the need for validated, scalable biomonitoring tools and field-deployable detection systems.

Because of the danger to human health, most countries have regulatory standards for the maximum allowable level of aflatoxin in food products and animal feed. Despite these regulatory standards, human exposure to aflatoxin B_1_ remains high, particularly in parts of Africa, Asia, and Latin America. Even in the United States, where human aflatoxin B_1_ exposure is low overall [103], aflatoxin exposure is much higher in certain regions, such as southern Texas, where 66% of the population around San Antonio had detectable levels of aflatoxin–albumin adducts [106]. This exposure and the associated health consequences highlight the need for improved strategies to reduce aflatoxin exposure. Global commerce underscores the global nature of the problem of aflatoxin contamination, even in countries with testing standards. Furthermore, climate change is likely to have a cross-cutting role in driving the geographic expansion of aflatoxin risk.

Multiple approaches are available for aflatoxin prevention and decontamination. In addition, modern advances, such as use of AI algorithms on mobile devices, could provide important breakthroughs in this area. Reducing exposure to aflatoxins requires an integrated One Health approach across the entire food system.

## Figures and Tables

**Table 1:** Prevalence of circulating aflatoxin–albumin adducts in studies with samples collected in 2010 or later.

Reference	Continent	Country	Age	Year of Blood Collection	N	Assay	Prevalence
Hoffmann 2018 [69]	Africa (East)	Kenya	Average 22 months	2015–2016	798	HPLC	100%
Lauer 2019 [76]	Africa (East)	Uganda	18–45 years	2017	220	HPLC	100%
Nabwire Wangia-Dixon 2020 [71]	Africa (East)	Kenya	6–12 years	2018	746	HPLC	100%
Kinyenje 2023 [73] *	Africa (East)	Tanzania	0.42–55 years	2019	26	LC-MS/MS	100%
Mekuria 2023 [67] *	Africa (East)	Ethiopia	Median 35 years	2020–2021	253	ELISA	64%
Osoro 2024 [70] *	Africa (East)	Kenya	Mean 29.3	2017–2019	250	LC-MS/MS	40%
Mshanga 2025 [72]	Africa (East)	Tanzania	6–24 months	2022	369	ELISA	70%
Tong 2025 [77]	Africa (East)	Uganda	Mean 33.2 years	2020–2021	114	HPLC	100%
Watson 2018 [56]	Africa (West)	The Gambia	18 months	2010	309	ELISA	99%
Xu 2021 [57]	Africa (West)	The Gambia	52 weeks	2012–2013	331	ELISA	98%
Chen 2022 [88]	Asia (East)	China (Guangxi)	Mean 28 years	2016–2017	320	ELISA	100%
Zhong 2024 [89]	Asia (East)	China (Guangxi)	Mean 30.1	2021–2022	126	HPLC-MS/MS	100%
Mitchell 2017 [83]	Asia (South)	Nepal	36 months	2013–2015	85	IDMS	89%
Mahfuz 2021 [79]	Asia (South)	Bangladesh	36 months	2010–2014	196	IDMS	62%
Ashraf 2022 [85]	Asia (South)	Pakistan	1–11 years	2020	238	HPLC-FLD	100%
Lamichhane 2022 [84] *	Asia (South)	Nepal	12 months	2016–2020	1329	HPLC-FLD	81%
Yadav 2025 [81] *	Asia (South)	India (Chandigarh)	Median 41 years	2021–2022	100	ELISA	81%
Shukla 2025 [82] *	Asia (South)	India (Jaipur)	Median 34–51 years	2021–2024	112	ELISA	47%
Kroker-Lobos 2019 [107]	Central America	Guatemala	Median 54	2016	439	IDMS	100%
Monge 2023 [105]	North America	Mexico (Veracruz, Chiapas, Tamaulipas, Campeche, Yucatán)	≥40 years (median 55)	2018–2019	952	IDMS	92%
Nogueira 2015 [108] *	South America	Chile (Santiago, Concepción, Temuco)	37–79 years	2012–2013	76	IDMS	21%

* Controls only. Abbreviations: HPLC, High-performance liquid chromatography; LC-MS/MS, Liquid chromatograph–tandem mass spectrometry; ELISA, Enzyme-linked immunosorbent assay; HPLC-MS/MS, High-performance liquid chromatography–tandem mass spectrometry; IDMS, Isotope dilution mass spectrometry; HPLC-FLD, High-performance liquid chromatography–fluorescence detector.

**Table 2:** Associations between detectable versus undetectable circulating aflatoxin B_1_–lysine adducts and gallbladder cancer.

Reference	Study Years	Location	Study Design	N Cases	N Controls	Assay	Specimen Type	Covariates	Control Type	OR	95% CI
Nogueira 2015 [108]	2012–2013	Santiago, Concepción, and Temuco, Chile	Community-based case–control	36	47	Isotope dilution mass spectrometry	Plasma	Weekly ají rojo consumption	Community controls	13.2	4.3–47.9
				36	29				Gallstone patients	9.4	2.8–37.2
Koshiol 2017 [95]	1997–2001	Shanghai, China	Population-based case–control	209	250	Isotope dilution mass spectrometry	Plasma	Age, sex	Gallstone patients	2.7	1.7–4.3
Yadav 2025 [81]	2021–2022	Chandigarh, India	Hospital-based case–control	51	100	ELISA	Serum	Age, sex, urban/rural, SES	Gallstone & normal *	6.8	1.3–35.7
Shukla 2025 [82]	2021–2024	Jaipur, India	Hospital-based case–control	45	57	ELISA	Serum	Age, sex	Gallstone patients	4.1	1.7–9.8
				45	55				Liver donors	16.8	4.0–70.2
Koshiol 2024 [96]	1986–1989	Shanghai, China	Case–control nested within residential cohort	84	168	Isotope dilution mass spectrometry	Serum	Age at sample collection, smoking, alcohol, BMI, education	Cohort participants †	2.0	1.0–3.9

* “Normal” in this study consists of patients with dyspeptic symptoms but without ultrasound-identified gallbladder disease. † Cohort participants who remained free of cancer and alive at the time of gallbladder cancer diagnosis of the index case.

**Table 3:** Maximum permitted levels of aflatoxins in food and milk in selected regulatory regions [23,237].

Region	AFB_1_ Limit (µg/kg)	Total Aflatoxins (µg/kg)	AFM_1_ in Milk (µg/kg)
Europe	2–12	4–15	0.025–0.05
United States	20	20	0.5
India	30	Not specified	0.5
China	0.5–20	-	0.5
Brazil	1–20	20	0.5
East Africa	5	10	0.5

Abbreviations: AFB_1_, aflatoxin B_1_; AFM_1_, aflatoxin M_1_.

## Data Availability

No data was generated. All data are publicly available.

## Supplementary Materials

The following supporting information can be downloaded at: https://www.mdpi.com/article/10.3390/toxins18020090/s1. Table S1: Worldwide prevalence of detectable circulating aflatoxin–albumin/aflatoxin lysine adducts.

## Abbreviations

The following abbreviations are used in this manuscript:

NHANES	National Health and Nutrition Examination Survey
HPLC	High-performance liquid chromatography
LC-MS/MS	Liquid chromatograph–tandem mass spectrometry
ELISA	Enzyme-linked immunosorbent assay
HPLC-MS/MS	High-performance liquid chromatography–tandem mass spectrometry
IDMS	Isotope dilution mass spectrometry
HPLC-FLD	High-performance liquid chromatography–fluorescence detector
RR	Relative risk
CI	Confidence interval
HBV	Hepatitis B virus
OR	Odds ratio
HCC	Hepatocellular carcinoma
GBD	Gallbladder cancer
ABC	Adenosine triphosphate-binding cassette
EHBDC	Extrahepatic bile duct cancer
SPIR	Standardized proportional incidence ratio
IHBDC	Intrahepatic bile duct cancer
SMR	Standardized mortality ratio
CYP450	Cytochrome 450
AFBO	Aflatoxin B_1_-exo 8,9-epoxide
HCV	Hepatitis C virus
CAP	Cold atmospheric plasma
AI	Artificial intelligence
NIH	National Institutes of Health
