# Supplementary material for: Aflatoxins and Human Health: Global Exposure, Disease Burden, and One Health Strategies
Source: Toxins (Basel). 2026 Feb 10;18(2):90. doi: 10.3390/toxins18020090 (PMC12944657; doi:10.3390/toxins18020090)
Supplement: Supplementary file 1 [file toxins-18-00090-s001.zip › toxins-4114947-supplementary.pdf]

Supplementary materials: Aflatoxins and Human Health: Global Exposure, Disease Burden, and One Health Strategies

Jill Koshiol, Amit Yadav, John D. Groopman and Usha Dutta

Supplemental Table S1. Worldwide prevalence of detectable circulating aflatoxin-albumin/aflatoxin lysine adducts

Reference	Continent	Country	Age	Year of blood collection	N	Assay	Prevalence
Gong 2004 [49]	Africa (West)	Benin	16-37 years	2001	200	ELISA	100%
Gong 2003 [50]	Africa (West)	Benin & Togo	<1-5 years	NR	479	ELISA	99%
Wild 1993 [51]*	Africa (West)	The Gambia	3-8 years	1988	390	ELISA	98%
Turner 2000 [53]	Africa (West)	The Gambia	3-4 years	1990-1991	444	ELISA	100%
Wild 2000 [54]	Africa (West)	The Gambia	Median 20/mean 24 years	1992	357	ELISA	99.7%
Turner 2003 [52]	Africa (West)	The Gambia	6-9 years	1989-1991	466	ELISA	93%
Turner 2007 [55]	Africa (West)	The Gambia	NR	1999-2000	119	ELISA	100%
Castelino 2014 [58]	Africa (West)	The Gambia	Mean 28.9 years	NR	134	ELISA	100%
Watson 2018 [56]	Africa (West)	The Gambia	18 months	2010	309	ELISA	99%
Xu 2021 [57]	Africa (West)	The Gambia	52 weeks	2012-2013	331	ELISA	98%
Diallo 1995 [59]	Africa (West)	Guinea	9-80 years, mean 39.7	1993	70	ELISA	94%
Turner 2002 [60]	Africa (West)	Guinea	NR	1996/1998	309	ELISA	82%

Turner 2005 [61] †	Africa (West)	Guinea	Mean 33.7 years	1999-2000	300	ELISA	98%
Turner 2005 [62]	Africa (West)	Guinea	2-5 years	2002	124	ELISA	96%
McGlynn 1995 [66]	Africa (West)	Ghana	Adults	NR	49	ELISA	51%
Jolly 2006 [63]	Africa (West)	Ghana	19-86 years	2002	162	RIA	100%
Jolly 2011 [65]	Africa (West)	Ghana	Mean 39 years	NR	314	RIA	99.7%
Shuaib 2012 [64]	Africa (West)	Ghana	Mean 27 years	2006	755	HPLC	100%
Wild 1993 [51]*	Africa (West)	Senegal	2 years	1988	29	ELISA	100%
Mekuria 2023 [67] †	Africa (East)	Ethiopia	Median 35 years	2020-2021	253	ELISA	64%
Chen 2018 [68]	Africa (East)	Tanzania	24 months	2009-2011	88	IDMS	72%
Wild 1993 [51]*	Africa (East)	Kenya	30 children aged 1-9 years; 61 adults	1988-1989	91	ELISA	47%
Hoffmann 2018 [69]	Africa (East)	Kenya	Average 22 months	2015-2016	798	HPLC	100%
Nabwire Wangia-Dixon 2020 [71]	Africa (East)	Kenya	6-12 years	2018	746	HPLC	100%
Osoro 2024 [70] †	Africa (East)	Kenya	Mean 29.3	2017-2019	250	LC-MS/MS	40%
Shirima 2013 [75]	Africa (East)	Tanzania	12-22 years	NR	146	ELISA	84%
Shirima 2015 [74]	Africa (East)	Tanzania	18-26 months	NR	143	ELISA	99%
Kinyenje 2023 [73] †	Africa (East)	Tanzania	0.42-55 years	2019	26	LC-MS/MS	100%
Mshanga 2025 [72]	Africa (East)	Tanzania	6-24 months	2022	369	ELISA	70%
Tong 2025 [77]	Africa (East)	Uganda	Mean 33.2 years	2020-2021	114	HPLC	100%
Lauer 2019 [76]	Africa (East)	Uganda	18-45 years	2017	220	HPLC	100%
Turner 2008 [78]†	Africa (North)	Egypt	37-73 years	1999-2004	24	ELISA	100%

Mahfuz 2021 [79]	Asia (South)	Bangladesh	36 months	2010-2014	196	IDMS	62%
Anitha 2014 [80]	Asia (South)	India (Hyderabad)	36-61 years	2009-2010	238	ELISA	16%
Yadav 2025 [81] †	Asia (South)	India (Chandigarh)	Median 41 years	2021-2022	100	ELISA	81%
Shukla 2025 [82] †	Asia (South)	India (Jaipur)	Median 34-51 years	2021-2024	112	ELISA	47%
Mitchell 2017 [83]	Asia (South)	Nepal	36 months	2013-2015	85	IDMS	89%
Lamichhane 2022 [84] †	Asia (South)	Nepal	12 months	2016-2020	1329	HPLC-FLD	81%
Ashraf 2022 [85]	Asia (South)	Pakistan	1-11 years	2020	238	HPLC-FLD	100%
Gan 1988 [86]	Asia (East)	China (Guangxi)	NR	NR	42	RIA	100%
Wild 1993 [51]	Asia (East)	China (Guangxi)	NR	NR	93	ELISA	70%
Wang 2001 [99]	Asia (East)	China (Guangxi)	25-60 years	1999	29	RIA + HPLC-FLD	100%
Chen 2022 [88]	Asia (East)	China (Guangxi)	Mean 28 years	2016-2017	320	ELISA	100%
Zhong 2024 [89]	Asia (East)	China (Guangxi)	Mean 30.1	2021-2022	126	HPLC-MS/MS	100%
Tao 2005 [90]	Asia (East)	China (Guangxi & Sichuan)	NR	2001	402	ELISA	100%
Yu 1995 [87]	Asia (East)	China (Haimen)	NR	NR	52	HPLC	71%
Xue 2019 [91] †	Asia (East)	China (Huaian)	Mean 61.9	2006-2007	205	HPLC-FLD	69%

Wang 1996 [92]	Asia (East)	China (Qidong)	>18 years	1993	600	RIA + HPLC-FLD	99.8%
Lu 1998 [93] †	Asia (East)	China (Qidong)	20-60 years	1987-1997	150	RIA	71%
Szymanska 2009 [94] †	Asia (East)	China (Qidong)	30-59 years	1989	123	ELISA	68%
Yu 1995 [87]	Asia (East)	China (Shanghai)	NR	NR	45	HPLC	9%
Koshiol 2017 [95] †	Asia (East)	China (Shanghai)	34-74 years	1997-2001	250	IDMS	15%
Koshiol 2024 [96] †	Asia (East)	China (Shanghai)	42-65 years	1986-1989	168	IDMS	61%
Wild 1993 [51]	Asia (East)	China (Shandong)	NR	NR	69	ELISA	0%
Yu 1995 [87]	Asia (East)	China (Shandong)	NR	NR	69	HPLC	0%
Chu 2018 [97] †	Asia (East)	Taiwan	30-65 years	1991-1992	1943	ELISA	54%
Chen 2001 [98]	Asia (East)	Taiwan	13-15 years	1991	200	ELISA	95%
Parkin 1991 [100] †	Asia (Mainland Southeast)	Thailand	Mean 54.6	1987-1988	21	ELISA	5%
Srivatanakul 1991 [101] †	Asia (Mainland Southeast)	Thailand	Mean 49 years	NR	45	Unclear	18%
Hollstein 1993 [102]	Asia (Mainland Southeast)	Thailand	17-73 years	1987-1991	8	ELISA	13%
Wild 1993* [51]	Asia (Mainland Southeast)	Thailand	Adults	1988-1989	84	ELISA	13%
Wild 1993 [51]*	Europe (West)	France	30 children aged 6 months to 15 years; 14 adults	1981, 1982, 1988	44	ELISA	0%

Wild 1993 [51]*	Europe (Central)	Poland	14 children aged 14-15 years; 16 adults	1988	30	ELISA	0%
Leroy 2018 [104]	North America	Mexico (Tabasco, Veracruz, Oaxaca, and Puebla)	6-12 months	2005-2006	347	HPLC	99%
Monge 2023 [105]	North America	Mexico (Veracruz, Chiapas, Tamaulipas, Campeche, Yucatán)	≥40 years (median 55)	2018-2019	952	IDMS	92%
Schleicher 2013 [103]	North America	United States	≥12 years	1999–2000	1572	IDMS	1%
Xue 2021 [106]	North America	United States (Texas)	NR	2004-2014	1124	HPLC-FLD + LC-MS	66%
Kroker-Lobos 2019 [107]	Central America	Guatemala	Median 54	2016	439	IDMS	100%
Nogueira 2015 [108] †	South America	Chile (Santiago, Concepción, Temuco)	37-79 years	2012-2013	76	IDMS	21%

NR: not reported

*Dates and ages from Wild, Carcinogenesis 1990 [45]

†Controls
